# Evaluation of Mental Health Profiles of Healthcare Workers in Northern Saudi Arabia: A Cross-Sectional DASS-21 Study with Implications for Prevention and Interdisciplinary Care

**DOI:** 10.3390/healthcare14081101

**Published:** 2026-04-20

**Authors:** Ahmed M. Alhuwaydi, Oqab Ahmed F. Alsharari, Abdulelah Faisal A. Alfandi, Ahmed Meshal H. Alorayyidh, Abdulrahman Yousef A. Alfayyadh, Ashokkumar Thirunavukkarasu, Aliyah Muteb Al-Ruwaili

**Affiliations:** 1 Department of Internal Medicine, Division of Psychiatry, College of Medicine, Jouf University, Sakaka 72388, Saudi Arabia; 2 College of Medicine, Jouf University, Sakaka 72388, Saudi Arabia; dr.oqabaf@gmail.com (O.A.F.A.); abdulelahalfandi@gmail.com (A.F.A.A.); a7med8477@gmail.com (A.M.H.A.); abdulrahmanyuniversity@gmail.com (A.Y.A.A.); 3 Department of Family and Community Medicine, College of Medicine, Jouf University, Sakaka 72388, Saudi Arabia; ashokkumar@ju.edu.sa; 4 Department of Public Health, Ministry of Health, Sakaka 73244, Saudi Arabia

**Keywords:** mental health, healthcare workers, depression, anxiety, stress, prevention, interdisciplinary care, Saudi Arabia

## Abstract

Background and objectives: Mental health assessment of healthcare workers (HCWs) is essential to inform prevention-oriented policies and interdisciplinary support strategies to strengthen HCWs’ mental health and optimize patient care. Therefore, the present study assessed mental health status and associated factors of HCWs using the DASS-21. Methods: Using a cross-sectional study design and the standardized DASS-21 questionnaire, we assessed the mental health status of HCWs of different categories from various healthcare settings of northern Saudi Arabia. A binomial logistic regression analysis was performed to examine the factors associated with each DASS-21 domain. Finally, Spearman’s correlation test was done to find the correlation across the domains. Results: Of the 385 participants, some forms of depression, anxiety, and stress were found in 49.6%, 49.4%, and 39.0% of the participants, respectively. Extremely severe symptoms were observed in depression and anxiety (9.6% each), and the lowest were observed for stress (3.9%). Depression was significantly associated with female gender (*p* = 0.017) and being single (*p* = 0.043), while anxiety was associated with nurses (*p* = 0.002) and non-Saudi nationality (*p* = 0.037). Stress was higher among HCWs working in specialty hospitals (*p* = 0.045) and lower among those aged > 40 years (*p* = 0.003). Furthermore, a positive correlation was noted within each DASS-21 domain (*p* < 0.001). Conclusions: Given the high prevalence of mental health issues, the relevant authorities should consider implementing preventative measures, including regular screening, psychoeducation workshops, interdisciplinary care, and proper referral pathways for the HCWs who screen positive for any of the mental health domains.

## 1. Introduction

Mental health challenges among working populations represent a significant global concern [1,2,3]. According to the World Health Organization (WHO), mental health conditions among working populations are associated with major productivity losses worldwide [3]. Healthcare workers (HCWs) are at special risk. Their occupations are exposed to chronic stress stimuli, which include high workloads, emotional components, extended working hours, and exposure to suffering and crisis situations. This exposure has been associated with high levels of depression, anxiety, stress, and burnout relative to the general population [4,5,6]. Poor mental health of HCWs is not only a humanitarian issue but also a concern that could create threats to patient safety, causing a correlation with a higher number of medical errors and poor care quality, alongside employee absences and turnover [7,8,9].

Regarding tools used for assessing mental health well-being, numerous standardized tools have been used worldwide to evaluate psychological well-being in healthcare professionals [10,11,12]. For example, the Patient Health Questionnaire—9 (PHQ-9) tool, which is a short and reliable tool available to measure depression [10] and anxiety, is frequently measured with the Generalized Anxiety Disorder—7 (GAD-7) tool [11]. Stress and burnout have often been assessed with the Perceived Stress Scale (PSS) [12] and the Maslach Burnout Inventory (MBI) [13], respectively. The Depression Anxiety Stress Scale (DASS) and the DASS-21 offer a psychometrically validated framework for assessing depression, anxiety, and stress simultaneously rather than as separate constructs. This multidimensional structure has supported its suitability for evaluating psychological symptoms in HCW populations [14].

The Saudi healthcare system has grown tremendously in the last decade [15]. However, the growth has come with a bigger burden on HCWs. Work-related stress factors, such as work pressure, irregular working hours, and a lack of psychological support, contribute to psychological pressure on employees. A recent study (2025) by Alharbi SH et al [16]. from the Qassim (central) region reported that a very high proportion of HCWs had deficient mental well-being. Their study also revealed that the prevalence of depression was higher among male participants [16]. Another 2025 study also revealed high levels of stress, anxiety, and depression among the physicians [17]. Despite these insights, most studies are limited in geographical scope or one professional category and are mostly centered in urban and major provinces.

The northern provinces of Saudi Arabia have received minimal research on HCW mental health status. The northern regions are unique in location, HCW distribution, sociocultural aspects, including greater distance from major urban centers, potentially more limited access to specialized mental health support services, and contextual sociocultural factors that may influence help-seeking. Therefore, challenges faced by these regions cannot be similar to those of other main cities in Saudi Arabia. To date, limited data and comprehensive assessments from northern Saudi Arabia have simultaneously evaluated depression, anxiety, and stress among all types of HCWs from different healthcare settings in northern Saudi Arabia.

Therefore, the present study assessed depression, anxiety, and stress among HCWs in northern Saudi Arabia using the DASS-21 and examined factors associated with these psychological outcomes. Furthermore, we hypothesized that symptoms of depression, anxiety, and stress would be prevalent among HCWs in northern Saudi Arabia and would vary according to selected demographic and work-related factors.

## 2. Materials and Methods

### 2.1. Study Design

The present cross-sectional study was conducted from October 2025 to December 2025. The reporting of this cross-sectional study was guided by the Strengthening the Reporting of Observational Studies in Epidemiology (STROBE) statement [18].

### 2.2. Study Setting

The study was conducted in Aljouf province, Saudi Arabia. This province is located in northern Saudi Arabia and comprises several healthcare facilities, including primary healthcare centers (PHCs) and general and specialty hospitals. The study included all HCWs involved in direct patient care. It included Saudis and all other nationals from various healthcare settings. We excluded HCWs on vacation, newly joined HCWs (first 6 months), trainees (students and interns), those involved solely in administrative work, and those unwilling to participate.

### 2.3. Sampling Process

The total eligible HCW population in the study region was 9675. The sample size was calculated using the online sample size calculator that uses the principles of Cochran’s formula (n = z^2^ pq/e^2^) [19]. Here, we used a 95% confidence interval with a 5% margin of error. Considering the evaluation of three domains from different HCWs from multiple settings, 50% of the expected proportion (*p*) of maximum participants was required to get a valid conclusion. Applying all these values, it was concluded that 384 HCWs were required for this study. To get the necessary participants, we used a convenience sampling method. In this method, participants were recruited from multiple healthcare facilities based on feasibility and access considerations. The data collectors aimed to get responses across different healthcare centers and HCW cadres to enhance representativeness.

### 2.4. Recruitment Strategies and Ethical Considerations

The research team adhered to the principles of the Declaration of Helsinki. Approval was obtained from the Jouf University Ethics Committee (approval no: 11291, dated: 24 September 2025). In addition to permissions from the relevant health cluster and healthcare facility administrators, the data collectors visited the facilities in person and invited eligible HCWs to participate. Participation was voluntary and anonymous, and informed consent was obtained at the start of the survey. Data were collected directly on the researcher’s tablets or smartphones using a Google Form made with the Depression, Anxiety, and Stress Scale—21 (DASS-21) items. The English version of the DASS-21 was used in the present study, as the participants were HCWs and were able to complete the instrument in English. Each participant completed the form privately, and no names or identifiers were recorded. To ensure diversity within convenience sampling, efforts were made to include individuals from diverse professions, departments, and shifts.

### 2.5. Data Collection Instrument

The DASS is a comprehensive instrument that measures three related aspects of mental health—depression, anxiety, and stress—within one survey. The full DASS comprises 42 items, and the commonly used DASS-21 is a 21-item version (7 items per subscale) that has been shortened to reduce participant burden [14]. Recent validation works in HCW samples have demonstrated that the DASS-21 is a reliable and valid screening tool for all three symptom domains among HCWs [20,21]. The depression subscale (items 3, 5, 10, 13, 16, 17, and 21) assessed hopelessness, lack of interest, low mood, and low self-esteem; the anxiety subscale (items 2, 4, 7, 9, 15, 19, and 20) measured autonomic arousal, situational anxiety, and fear responses; and the stress subscale evaluated (items 1, 6, 8, 11, 12, 14, and 18) chronic tension, irritability, difficulty relaxing, and impatience. The HCWs responded to each item from 0 (did not apply at all) to 3 (applied most of the time). Subscale scores were summed and then multiplied by 2 to make them comparable with the original DASS-42. Severity was categorized as normal, mild, moderate, severe, or extremely severe for each domain. In the present sample, the DASS-21 demonstrated acceptable-to-good internal consistency, with Cronbach’s alpha coefficients of 0.71 for the depression subscale, 0.79 for the anxiety subscale, 0.83 for the stress subscale, and 0.76 for the overall scale.

### 2.6. Data Analysis

We exported the Excel sheet (downloaded from Google Form) to SPSS version 21.0 for data curation and analysis. The descriptive data are presented as frequencies, percentages, means, medians, and other relevant measures of dispersion such as standard deviations (SD) and interquartile range (IQR). The present study’s data did not meet the normality assumption based on visual inspection of Q–Q plots and the Shapiro–Wilk test results. Therefore, we performed Spearman’s correlation test to find the correlation across the domains. For multivariable analysis using the binomial logistic regression, the original categories were collapsed into a binary outcome (normal–mild vs moderate–extremely severe). Because the DASS-21 outcomes were analyzed as binary severity categories rather than continuous scores, binomial logistic regression was considered more appropriate than linear regression. This approach was used to distinguish participants with at least moderate symptom burden, which is commonly treated as a more clinically meaningful threshold in DASS-21-based analyses, while also supporting stable regression modelling across categories. Accordingly, the binary outcomes were defined using standard DASS-21 severity thresholds as follows: depression, normal–mild (0–13) vs. moderate–extremely severe (≥14); anxiety, normal–mild (0–9) vs. moderate–extremely severe (≥10); and stress, normal–mild (0–18) vs. moderate–extremely severe (≥19) [22,23,24]. Binomial regression was performed using the enter method, whereby the selected independent variables were entered simultaneously into the model. Multicollinearity among independent variables was assessed using the variance inflation factor (VIF) and tolerance statistics, and no evidence of problematic multicollinearity was observed. In the regression analysis, we obtained adjusted odds ratios (AORs) and 95% confidence intervals (CIs) to determine the factors associated with each domain. A *p*-value of less than 0.05 was set as a statistically significant value.

## 3. Results

During data collection, 433 eligible HCWs were approached for participation (response rate: 88.9%). Of the 385 respondents, 51.7% were female. Most were aged 30–40 years (mean ± SD = 34.94 ± 8.6), married (62.9%), and held a minimum of a bachelor’s degree (74.0%). The majority were Saudi nationals (71.7%), worked in general hospitals (39.5%), were nurses (39.5%), and had over 10 years of work experience (38.2%) (Table 1).

Item-wise responses to the DASS-21 scale are presented in the Supplementary Material (please find more details in Supplementary Table S1).

Table 2 summarizes the DASS-21 subscale scores among the HCWs. Overall, stress showed the highest central tendency (mean 6.69 ± 5.04; median 6 [IQR 7]), followed by depression (mean 5.56 ± 5.12; median 4 [IQR 7]) and anxiety (mean 4.23 ± 3.83; median 4 [IQR 6]).

Table 3 shows that about half of HCWs were in the normal range for depression (50.4%) and anxiety (50.6%), while stress was more often normal (61.0%). Extremely severe symptoms were observed in depression and anxiety (9.6% each), and the lowest were observed for stress (3.9%).

Of the analyzed scores, a moderate positive correlation was found between depression and anxiety (Spearman’s rho [ρ] = 0.512, *p* < 0.001) and depression and stress (ρ = 0.585, *p* < 0.001). A strong positive correlation was noted between anxiety and stress (ρ = 0.726, *p* < 0.001) (Table 4).

Table 5 shows the factors associated with the depression domain. Of the analyzed data, higher odds of moderate-to-extremely severe symptoms of depression were observed among females (ref: males, AOR = 2.36, 95% CI = 1.67–3.03, *p* = 0.017), single people (ref: married, AOR = 1.48, 95% CI = 1.07–1.95, *p* = 0.043), nurses (ref: doctors, AOR = 3.85, 95% CI = 2.15–5.30, *p* = 0.001), and those working in critical care wards for inpatient care (ref: outpatient clinic, AOR = 2.17, 95% CI = 1.79–3.61, *p* = 0.007). Significantly lower odds of having moderate-to-extremely severe symptoms of depression were observed among pharmacists (ref: doctors, AOR = 0.58, 95% CI = 0.31–0.84, *p* = 0.001).

Table 6 shows the factors associated with the anxiety domain. Significantly higher odds of having anxiety symptoms were observed among females (ref: males, AOR = 1.59, 95% CI = 1.05–2.07, *p* = 0.041), non-Saudi nationals (ref: Saudis, AOR = 2.63, 95% CI = 1.42–3.34, *p* = 0.037), HCWs working in specialty hospitals (ref: PHC, AOR = 1.85, 95% CI = 1.23–2.62, *p* = 0.037), nurses (ref: doctors, AOR = 1.71, 95% CI = 1.29–3.73, *p* = 0.002), and those working in critical care wards in inpatient care (ref: outpatient clinic, AOR = 2.06, 95% CI = 1.31–3.48, *p* = 0.005). Significantly lower odds of having moderate-to-extremely severe symptoms of depression were observed among those aged more than 40 years (ref: less than 30 years, AOR = 0.67, 95% CI = 0.36–0.90, *p* = 0.006).

The factors associated with the stress symptoms domain are presented in Table 7. Of the data analyzed, significantly higher odds of having stress symptoms were noted among those working in a specialty hospital (ref: PHC, AOR = 1.76, 95% CI = 1.04–2.71, *p* = 0.045), and significantly lower odds of having stress symptoms were found among those aged more than 40 years (ref: less than 30 years, AOR = 0.58, 95% CI = 0.41–0.69, *p* = 0.003).

## 4. Discussion

Mental health challenges among HCWs can undermine both workforce well-being and patient care. Therefore, it is essential to identify the current mental health status among them, especially in unexplored regions. This study identified the prevalence of depression, anxiety, and stress symptoms in HCWs in northern Saudi Arabia using the DASS-21 and factors associated with each domain. Overall, a considerable proportion of HCWs reported symptoms across all three domains, with depression and anxiety affecting nearly half of the participants, while stress was comparatively lower but still substantial. In the adjusted analysis, several demographic and workplace factors were associated with these psychological outcomes, including gender, nationality, professional category, work setting, workplace type, and age.

The present study revealed that approximately half of the HCWs reported at least mild depressive symptoms, and about one-third of the participants had experienced moderate-to-extremely severe levels of depressive symptoms. When comparing the present study’s prevalence of depression rates, there is a wide range of variations reported in the recent literature across studies, both at the regional and international levels [25,26,27]. These variations are even found across Middle-Eastern countries [17,26,28]. For example, a recent Egyptian study stated that the HCWs in their study had varying degrees of depression symptoms, ranging from mild (26.1%) to severe (43.1%) [28]. In contrast, a global meta-analysis of 65 studies reported a lower proportion of pooled prevalence of depression; a level of about 22% was found among the HCWs. Even in their study, the highest proportion of depression was found to be among Middle-Eastern HCWs [29]. Interestingly, an Iranian study reported a lower level of depressive symptoms among HCWs that were assessed by the DASS-21 scale compared to the present study [26]. The substantial burden of depressive symptoms among HCWs suggests the importance of proactive mental health strategies in the healthcare system. Furthermore, these varying patterns of prevalence indicate that these symptoms vary across regions and over time. Routine screening programs may be considered as part of preventive mental health strategies for HCWs; however, longitudinal research is needed to evaluate their effectiveness and potential impact over time.

In the present study, higher odds of moderate-to-extremely severe depression were observed in female staff, single individuals, nurses, and those working in critical care in inpatient units. Conversely, pharmacists showed significantly lower odds of depression. Similar to the prevalence pattern, the factors associated with depressive symptoms also vary across studies [26,30,31,32]. Nonetheless, the observed higher prevalence among female staff and those working in critical care units aligns with broad trends reported in the literature [31,33]. However, not all findings were fully consistent with earlier reports. For example, while some previous Saudi studies have reported higher depressive symptoms among male HCWs, our study observed higher odds among female staff. This difference may reflect contextual variations in workforce composition, workplace demands, and regional service organization in northern Saudi Arabia. The lower levels of depressive symptoms among pharmacists could possibly be due to less direct exposure to traumatic patient outcomes or more stable work routines. Our findings are supported by Kakemam et al. In their study (2024), a lower proportion (42.6%) of primary care HCWs had symptoms of depression [26]. These identified factors in the present study suggest that each professional role and setting may be associated with distinct psychological risks, which may be related to the nature of patient interaction and workplace pressures. These findings may support consideration of interdisciplinary care models as a potentially relevant approach. For instance, embedding mental health professionals (psychologists or psychiatrists) within healthcare teams by conducting targeted debriefings and counselling with high-risk HCWs (like critical care), normalizing discussions about emotional struggles, and providing early therapy to those in need could be considered. Furthermore, the recent literature suggests that digital staff support interventions and GenAI-based approaches may have potential to support psychological well-being among HCWs [34,35].

The present research results regarding anxiety prevalence are lower than the figures reported at the height of the pandemic in the region. For example, AlAteeq et al. in 2020 found that 51.4% of HCWs met criteria for generalized anxiety disorder during the first COVID wave [36], and in Egypt, nearly 90% of HCWs surveyed had some degree of anxiety (with 18.5% being severe) [37]. Similar to the present study, lower levels of anxiety symptoms were found in a recent study (2024) from China by Lu J et al. In their study, about 47% of the study participants showed anxiety symptoms [38]. In contrast, another study from Saudi Arabia that was conducted in 2025 by Alharbi SH et al. stated that a very high proportion of the HCWs had experienced generalized anxiety disorders [16]. These variations in prevalence across studies may be due to sociocultural factors, organizational support, participants’ demographic characteristics, included work settings, and the assessment tool used. The present study included all HCWs from various nationalities working across multiple healthcare settings.

Our study findings revealed that anxiety symptoms were significantly higher among certain variables, such as females, those working in specialty hospitals, and nurses. Another important finding identified in the present study was that the odds of having moderate-to-extremely severe anxiety were significantly higher among expatriates (non-Saudi nationalities). This may be related to language barriers, differences in sociocultural environments, job insecurity, and separation from family and friends. Even though some studies identified differences in predictors [38,39,40], our findings are supported by previous studies among HCWs. For example, in a Saudi survey done by Shamsan et al., women scored higher on anxiety scales, and both groups of younger workers (<30 years and 30 to 39 years) had higher anxiety [41]. Higher anxiety among younger HCWs may be due to uncertainty and job-related worries, whereas older staff have accrued coping skills. Similarly, higher anxiety among HCWs in critical units may be associated with greater workload and exposure to more severe cases in such settings. The prominence of anxiety among expatriate HCWs also adds an important context-specific finding. In northern Saudi Arabia, regional workforce dependence and differences in social and professional support may partly explain this pattern. Our findings suggest numerous insights into the prevention and management of anxiety-related symptoms among HCWs. Given the high prevalence of anxiety symptoms, the relevant authorities may consider implementing preventative measures for anxiety reduction, including coping skills, psychoeducation workshops, interdisciplinary care, referral pathways for HCWs screened positive for anxiety, and implementation of WHO and CDC measures for HCW workplace well-being [42,43].

In this study, the stress domain had the lowest prevalence among the three DASS-21 facets, yet a sizable proportion of participants reported stress-related symptoms. In contrast to the other two domains, stress levels did not differ significantly by gender or profession in our sample. These results suggest that stress among HCWs is more consistently distributed across types of HCWs and may be more closely associated with structural workplace factors and that personal demographics may have a smaller influence [44,45]. This interpretation is also supported by previous studies suggesting that occupational stress among HCWs is often driven more by organizational and workplace conditions than by gender or professional category alone. Factors such as workload, staffing patterns, shift schedules, and institutional support have been identified as important contributors to stress across different HCW groups [46,47]. A recent meta-analysis (2025) by Alhassan et al., which focused primarily on Middle-Eastern countries, found that burnout (an effect of stress) is commonly found in countries such as Saudi Arabia [48]. Also, the present study found higher odds of stress among those working in specialty hospitals and lower odds among those in the higher age group. This suggests that, in this setting, stress may be more closely related to workplace structure than to broader demographic characteristics. Furthermore, the organizational stressors may be important targets for workplace-based stress reduction strategies [43,49]. Furthermore, from an interdisciplinary care model perspective, the concerned authorities may consider stress reduction facilities with relevant resources, such as physiotherapists or occupational therapists, along with stress reduction breaks during working hours to support HCWs’ use of such resources where feasible [50,51].

Our study found that all three domains of the DASS-21 were positively correlated, and this suggests an interlinkage between stress, anxiety, and depression with possible implications for both clinical practice and policy. Similar to the present study, a study conducted among HCWs by Emiral E et al. in 2023 using the DASS-21 scale reported a positive correlation among all three DASS-21 domains [52]. Therefore, screening for mental health profiles may not be restricted to a single domain. For instance, if an HCW screening for mental health is positive for one domain, they may warrant evaluation for all domains. Our study’s findings and implications are supported by previous studies, which have found an interlink between various mental health problems. Furthermore, mindfulness-based stress reduction training may alleviate mental health problems of HCWs [53,54].

The present study has several strengths. First, this study is one of the very few recent investigations that were conducted among diverse HCWs from various healthcare settings that assessed depression, anxiety, and stress simultaneously. Furthermore, the study used a standardized and validated tool (DASS-21) that enhances the comparability of the present study’s findings with the global literature. Third, the study focused on a relatively under-researched area in Saudi Arabia and provides evidence to help in understanding the mental health profiles of HCWs and their geographical variations. Nonetheless, the present study has limitations to consider when interpreting the findings. First, the DASS-21 is a screening tool and not a diagnostic instrument; thus, it cannot confirm clinical diagnoses of depression, anxiety, or stress. Furthermore, the psychological symptoms were assessed at a single time point, which may reflect temporary states rather than more persistent psychological conditions. In addition, collapsing DASS-21 severity categories into binary outcomes may have reduced distinctions between normal and mild symptoms, although this approach was used to identify HCWs with at least moderate symptom burden for regression analysis. Second, we used a cross-sectional study design, which cannot identify causal relationships with associated factors or within each domain. Next, we used convenience sampling to recruit the participants from a single region. Therefore, the present study’s findings may reflect regional and contextual characteristics of this setting, may not represent the mental health profiles of HCWs and may not be generalizable to other parts of Saudi Arabia. Moreover, online data collection through Google Forms may involve technical issues such as connection interruptions and perceived data security concerns. Finally, bias related to self-reported surveys cannot be ignored, including the possibility of underreporting or overreporting due to social desirability or individual response tendencies.

## 5. Conclusions

The present study found significant levels of symptoms of depression, anxiety, and stress among northern Saudi HCWs, according to the DASS-21. Given the high prevalence of mental health issues, the relevant authorities may consider implementing preventative measures for mental health issues, including regular screening, psychoeducation workshops, interdisciplinary care, and appropriate referral pathways for the HCWs who screen positive for any of the mental health domains. Next, the findings suggest the potential value of target-oriented mental health prevention programs due to the burden being high in certain groups and settings (e.g., specialty hospitals and inpatient/critical care settings). Moreover, these findings may help guide attention toward measures at the organizational level to mitigate modifiable workplace stressors, enhance early identification and referral pathways, and introduce models of interdisciplinary care to support the mental health of HCWs and ensure safe patient care. Finally, mixed-methods studies among HCWs could be conducted to explore the qualitative aspects of their mental health status, including through qualitative interviews or focus group discussions, and longitudinal follow-up studies could be considered to assess changes over time.

## Figures and Tables

**Table 1:** Background characteristics of the study participants (n = 385).

Variables	Frequency	Proportion (%)
Age (years) (mean ± SD)	34.94 ± 8.6	
Age group		
Less than 30	131	34.0
30 to 40	157	40.8
More than 40	97	25.2
Gender		
Male	186	48.3
Female	199	51.7
Marital status		
Married	242	62.9
Single	143	37.1
Education status		
Up to bachelor’s degree	285	74.0
Above bachelor’s degree	100	26.0
Nationality		
Saudi	276	71.7
Non-Saudi	109	28.3
Monthly income in SAR (1 USD = 3.75 SAR)		
Up to 10,000	165	42.9
More than 10,000	220	57.1
Work setting		
Primary health center (PHC)	110	28.6
General hospital	152	39.5
Specialty hospital	123	31.9
Healthcare worker (HCW) category		
Doctors	117	30.4
Nurses	152	39.5
Pharmacists	42	10.9
Lab technicians	39	10.1
Others	35	9.1
Work experience (years)		
Up to 5	130	33.8
5 to 10	108	28.1
More than 10	147	38.2
Workplace		
Outpatient clinic	186	48.3
Inpatients (non-critical)	80	20.8
Inpatients (critical wards)	119	30.9

**Table 2:** DASS-21 subscale scores among HCWs.

Subscale	Mean ± SD	Median (IQR)	Min–Max
Depression	5.56 ± 5.12	4 (7)	0–21
Anxiety	4.23 ± 3.83	4 (6)	0–16
Stress	6.69 ± 5.04	6 (7)	0–21

**Table 3:** Severity categories of depression, anxiety, and stress among HCWs (n = 385).

Severity Level	Depression n (%)	Anxiety n (%)	Stress n (%)
Normal	194 (50.4)	195 (50.6)	235 (61.0)
Mild	53 (13.8)	43 (11.2)	50 (13.0)
Moderate	74 (19.2)	80 (20.8)	39 (10.1)
Severe	27 (7.0)	30 (7.8)	46 (11.9)
Extremely severe	37 (9.6)	37 (9.6)	15 (3.9)

**Table 4:** Correlation between depression, anxiety, and stress subscale scores.

Pair of Subscales	Spearman’s Rho	*p*-Value
Depression vs. Anxiety	0.512	<0.001
Depression vs. Stress	0.585	<0.001
Anxiety vs. Stress	0.726	<0.001

**Table 5:** Multivariable binomial logistic regression analysis of factors associated with depression (n = 385).

Variable	Total	Depression		Adjusted Odds Ratio (AOR) (95% CI)	*p*-Value
		Normal to Mild (n = 247)	Moderate to Extremely Severe (n = 138)		
Age group					
Less than 30	131	82	49	Ref	
30 to 40	157	97	60	0.81 (0.59–2.33)	0.124
More than 40	97	68	29	0.92 (0.78–1.86)	0.590
Gender					
Male	186	125	61	Ref	
Female	199	122	77	2.36 (1.67–3.03)	0.001
Marital status					
Married	242	161	81	Ref	
Single	143	86	57	1.48 (1.07–1.95)	0.043
Education status					
Up to bachelor’s degree	285	182	103	Ref	
Above bachelor’s degree	100	65	35	0.77 (0.49–2.87)	0.285
Nationality					
Saudi	276	180	96	Ref	
Non-Saudi	109	67	42	0.85 (0.64–1.92)	0.572
Monthly income in SAR					
Up to 10,000	165	97	68	Ref	
More than 10,000	220	150	70	0.59 (0.36–3.12)	0.687
Work setting					
PHC	110	81	29	Ref	
General hospital	152	89	63	1.76 (0.91–3.47)	0.106
Specialty hospital	123	77	46	1.60 (0.94–2.63)	0.158
Healthcare worker (HCW) category					
Doctors	117	77	40	Ref	
Nurses	152	85	67	3.85 (2.15–5.30)	0.001
Pharmacists	42	33	9	0.58 (0.31–0.84)	0.001
Lab technicians	39	29	10	0.89 (0.63–3.06)	0.436
Others	35	23	12	1.52 (0.82–3.29)	0.253
Work experience (years)					
Up to 5	130	78	52	Ref	
5 to 10	108	68	40	1.86 (0.71–4.16)	0.739
More than 10	147	101	46	0.77 (0.59–2.87)	0.473
Workplace					
Outpatient clinic	186	127	59	Ref	
Inpatients (non-critical)	80	59	21	0.61 (0.43–2.58)	0.107
Inpatients (critical wards)	119	61	58	2.17 (1.79–3.61)	0.007

**Table 6:** Multivariable binomial logistic regression analysis of factors associated with anxiety (n = 385).

Variable	Total	Anxiety		AOR (95% CI)	*p*-Value
		Normal to Mild (n = 238)	Moderate to Extremely Severe (n = 147)		
Age group					
Less than 30	131	74	57	Ref	
30 to 40	157	94	63	1.38 (0.71–2.59)	0.204
More than 40	97	70	27	0.67 (0.36–0.90)	0.006
Gender					
Male	186	128	58	Ref	
Female	199	110	89	1.59 (1.07–2.05)	0.041
Marital status					
Married	242	154	88	Ref	
Single	143	84	59	1.71 (0.72–4.37)	0.496
Education status					
Up to bachelor’s degree	285	174	111	Ref	
Above bachelor’s degree	100	64	36	1.05 (0.60–2.67)	0.118
Nationality					
Saudi	276	175	101	Ref	
Non-Saudi	109	63	46	2.63 (1.42–4.34)	0.037
Monthly income in SAR					
Up to 10,000	165	100	65	Ref	
More than 10,000	220	138	82	1.19 (0.74–1.89)	0.427
Work setting					
PHC	110	76	34	Ref	
General hospital	152	95	57	0.75 (0.46–2.83)	0.194
Specialty hospital	123	67	56	1.85 (1.23–2.62)	0.037
HCW category					
Doctors	117	77	40	Ref	
Nurses	152	81	71	1.71 (1.29–3.73)	0.002
Pharmacists	42	31	11	0.92 (0.52–1.62)	0.813
Lab technicians	39	25	14	1.36 (0.65–2.85)	0.169
Others	35	24	11	0.48 (0.37–1.48)	0.319
Work experience (years)					
Up to 5	130	73	57	Ref	
5 to 10	108	68	40	1.51 (0.70–3.13)	0.088
More than 10	147	97	50	1.29 (0.67–2.45)	0.172
Workplace					
Outpatient clinic	186	124	62	Ref	
Inpatients (non-critical)	80	49	31	1.75 (0.87–3.65)	0.093
Inpatients (critical wards)	119	65	54	2.06 (1.31–3.48)	0.005

**Table 7:** Multivariable binomial logistic regression analysis of factors associated with stress (n = 385).

Variable	Total	Stress		AOR (95% CI)	*p*-Value
		Normal to Mild (n = 285)	Moderate to Extremely Severe (n = 100)		
Age group					
Less than 30	131	91	40	Ref	
30 to 40	157	115	42	0.94 (0.63–2.43)	0.120
More than 40	97	79	18	0.58 (0.41–0.79)	0.003
Gender					
Male	186	134	52	Ref	
Female	199	151	48	0.74 (0.51–2.66)	0.905
Marital status					
Married	242	184	58	Ref	
Single	143	101	42	1.99 (0.70–5.19)	0.707
Education status					
Up to bachelor’s degree	285	209	76	Ref	
Above bachelor’s degree	100	76	24	1.17 (0.55–3.32)	0.638
Nationality					
Saudi	276	202	74	Ref	
Non-Saudi	109	83	26	1.83 (0.88–3.01)	0.116
Monthly income in SAR					
Up to 10,000	165	118	47	Ref	
More than 10,000	220	167	53	0.86 (0.63–2.04)	0.173
Work setting					
PHC	110	94	16	Ref	
General hospital	152	103	49	1.14 (0.67–3.07)	0.449
Specialty hospital	123	88	35	1.76 (1.04–2.71)	0.045
HCW category					
Doctors	117	88	29	Ref	
Nurses	152	106	46	0.79 (0.53–2.48)	0.830
Pharmacists	42	32	10	0.38 (0.28–3.53)	0.549
Lab technicians	39	29	10	0.87 (0.61–2.79)	0.148
Others	35	30	5	0.97 (0.86–2.27)	0.100
Work experience (years)					
Up to 5	130	88	42	Ref	
5 to 10	108	78	30	1.73 (0.69–4.25)	0.893
More than 10	147	119	28	0.91 (0.67–2.66)	0.481
Workplace					
Outpatient clinic	186	149	37	Ref	
Inpatients (non-critical)	80	66	14	0.55 (0.36–2.20)	0.510
Inpatients (critical wards)	119	70	49	1.57 (0.63–3.83)	0.174

## Data Availability

The original contributions presented in this study are included in the article/Supplementary Material. Further inquiries can be directed to the corresponding author.

## Supplementary Materials

The following supporting information can be downloaded at: https://www.mdpi.com/article/10.3390/healthcare14081101/s1, Table S1: Item-wise responses to the DASS-21 (n = 385). Mental Health DASS 21 Data.
