# Supplementary material for: Evaluation of Mental Health Profiles of Healthcare Workers in Northern Saudi Arabia: A Cross-Sectional DASS-21 Study with Implications for Prevention and Interdisciplinary Care
Source: Healthcare (Basel). 2026 Apr 20;14(8):1101. doi: 10.3390/healthcare14081101 (PMC13116207; doi:10.3390/healthcare14081101)
Supplement: Supplementary file 1 [file healthcare-14-01101-s001.zip › Supplementary Table S1.pdf]

Supplementary Table S1. Item-wise responses to the DASS-21 (n = 385)

Item No.	Did not apply to me at all n (%)	Applied to some of the time n (%)	Applied to me to a good part of the time n (%)	Applied to me very much, or most of the time n (%)
1	102 (26.5)	158 (41.0)	83 (21.6)	42 (10.9)
2	183 (47.5)	143 (37.1)	44 (11.4)	15 (3.9)
3	161 (41.8)	132 (34.3)	67 (17.4)	25 (6.5)
4	234 (60.8)	97 (25.2)	49 (12.7)	5 (1.3)
5	168 (43.6)	132 (34.3)	66 (17.1)	19 (4.9)
6	158 (41.0)	135 (35.1)	74 (19.2)	18 (4.7)
7	221 (57.4)	119 (30.9)	43 (11.2)	2 (0.5)
8	124 (32.2)	137 (35.6)	80 (20.8)	44 (11.4)
9	187 (48.6)	140 (36.4)	41 (10.6)	17 (4.4)
10	193 (50.1)	113 (29.4)	60 (15.6)	19 (4.9)
11	153 (39.7)	145 (37.7)	64 (16.6)	23 (6.0)
12	124 (32.2)	154 (40.0)	84 (21.8)	23 (6.0)
13	163 (42.3)	135 (35.1)	63 (16.4)	24 (6.2)
14	168 (43.6)	127 (33.0)	74 (19.2)	16 (4.2)
15	239 (62.1)	106 (27.5)	36 (9.4)	4 (1.0)
16	166 (43.1)	125 (32.5)	64 (16.6)	30 (7.8)
17	225 (58.4)	98 (25.5)	44 (11.4)	18 (4.7)
18	178 (46.2)	130 (33.8)	59 (15.3)	18 (4.7)
19	205 (53.2)	128 (33.2)	45 (11.7)	7 (1.8)
20	216 (56.1)	128 (33.2)	35 (9.1)	6 (1.6)
21	217 (56.4)	98 (25.5)	46 (11.9)	24 (6.2)
